# The challenges of implementing earlier surgery for terminal ileal Crohn's disease—A qualitative study of the clinician's perspective

**DOI:** 10.1111/codi.70027

**Published:** 2025-02-11

**Authors:** Nilofer Husnoo, Jenna L. Morgan, Lynda Wyld, Alan J. Lobo, Steven R. Brown

**Affiliations:** ^1^ School of Medicine and Population Health University of Sheffield Sheffield UK; ^2^ Sheffield Teaching Hospitals NHS Foundation Trust Northern General Hospital Sheffield UK; ^3^ Doncaster and Bassetlaw Teaching Hospitals NHS Trust Doncaster Royal Infirmary Doncaster UK

**Keywords:** Crohn's disease, surgery, terminal ileitis

## Abstract

**Aim:**

Evidence suggests that earlier bowel resection may offer more stable remission in localized luminal terminal ileal (TI) Crohn's disease compared with ongoing medical therapy. Surgery is still considered late in the treatment pathway. The aim of this study was to understand the clinician's perspective on ‘early’ surgery by qualitatively exploring how clinicians make treatment‐related decisions.

**Method:**

Semistructured interviews with clinicians across the UK with an interest in inflammatory bowel disease (IBD) were undertaken using videoconferencing (February–November 2022). Inductive thematic analysis of interview transcripts was performed; 10% of the data were double‐coded. Data saturation was confirmed before stopping recruitment.

**Results:**

Participants included nine consultant surgeons, seven consultant gastroenterologists and seven specialist nurses (*n* = 23) from secondary care and tertiary referral centres. Five key themes were identified: timing of surgery in practice, barriers to timely surgery, factors influencing decision‐making, offering choice and the patient's perspective. A practice of exhausting medical options before considering surgery was commonly described. A lack of IBD specialists (especially surgeons), inadequate opportunities for multidisciplinary teamwork and long waiting lists for surgical clinics and theatre were cited as barriers to timely surgery. According to interviewees, patients prefer medical therapy over surgery; the most dreaded risk is thought to be that of a stoma.

**Conclusion:**

This study provides new insights into the barriers to earlier surgery for TI disease. Organizational barriers should be considered when designing local services. Collaborative multidisciplinary teamwork may allow clinicians to consider surgery sooner. A study investigating the patient perspective is warranted.

**What does this paper add to the literature?**

Surgery is still considered late in the treatment of isolated terminal ileal Crohn's disease despite the evidence in favour of earlier resection. This study highlights key barriers to surgery from the clinician's perspective, which should be considered when designing local inflammatory bowel disease services and when designing future trials.

## INTRODUCTION

Crohn's disease (CD) most commonly affects the terminal ileum (TI), with or without caecal involvement. This phenotype occurs in one third of patients [1]. With the increasing availability of effective medical therapies to treat CD, surgery tends to be considered only when medical therapy fails or complications arise, rather than as a primary option or an alternative to medical therapy. There is mounting evidence in favour of earlier bowel resection for isolated luminal ileocaecal CD. A recent systematic review showed that, compared with initial medical therapy, early bowel resection is associated with a reduced need for surgery at 5 years and for drug therapy [2].

The most significant study contributing to the evidence base is the LIR!C (Laparoscopic ileocaecal resection versus infliximab for terminal ileitis in Crohn's disease) trial [3]. Short‐term data suggested that surgery and infliximab produced equivalent quality of life scores, and that surgery was more cost‐effective [4]. At 5 years, three‐quarters of the surgical group avoided further biologicals and no patient required a second resection, while nearly half in the infliximab arm underwent surgery [5]. Accordingly, clinical guidelines support consideration of early surgery for CD localized to the terminal ileum [6, 7, 8].

The adoption of ‘early surgery’ into practice requires due consideration to be given to several factors. The definition of ‘early’ surgery varies across studies; ongoing medical therapy has been compared with surgery at different points in the course of the disease. The optimal timing of an operation that will produce the best outcomes is unknown. LIR!C is the only randomized controlled trial comparing early bowel resection with medical therapy. Further high‐quality studies comparing the two treatment modalities remain a research priority [9]. These trials are difficult to conduct, evidenced by the length of time taken to complete the LIR!C trial (7.5 years) and the early termination of other trials comparing these two treatment options due to recruitment difficulties [10, 11]. Most patients who declined participation in the LIR!C trial reportedly did so because of their personal preference for one treatment over the other [12].

While clinicians and researchers have indicated a need for a change in practice in favour of earlier surgery for localized luminal TI disease, no studies have explored whether this change has occurred, or how feasible it is, considering the issues encountered in a trial setting. An understanding of this is needed to identify potential facilitators or barriers to evidence‐based care and to design future trials appropriately. Clinicians are key stakeholders in the treatment decision‐making process. The aim of this study was to investigate their perspective on ‘early’ surgery for localized luminal ileocaecal CD, by qualitatively exploring how they make treatment‐related decisions and what they perceive the role of surgery to be.

## METHOD

This study received ethical approval from the University of Sheffield Research Ethics Committee (reference 044137). Written consent was obtained from study participants. The study has been reported according to the consolidated criteria for reporting qualitative research (COREQ) [13].

### Study design

Qualitative methodology was selected for this exploratory study as there is no existing literature investigating clinicians' treatment preferences for this patient cohort. An in‐depth understanding of participants' experiences and perspectives was required [14]. Interviews were semistructured, i.e. a predefined schedule was used to cover areas of interest, while allowing the interviewer and interviewee the freedom to discuss emergent themes as they arose [15].

Healthcare professionals (HCPs) from across the United Kingdom (UK) were eligible to participate if they were a consultant gastroenterologist, consultant colorectal surgeon or a clinical nurse specialist (CNS) with a claimed interest or expertise in inflammatory bowel disease (IBD) and working in the National Health Service (NHS). A purposive sample was recruited, based on job role, level of specialist IBD care provided at the participant's unit (tertiary referral centre versus secondary care) and geographical location to ensure a mix of experience and representation across the UK. Participants were recruited through social media and through the study team's contacts using a snowballing strategy (whereby participants helped with recruitment by identifying and contacting eligible candidates from their personal and professional networks [16]).

Recruitment stopped when data saturation was achieved. Previous work in the field showed that data saturation can be achieved with 15 interviews [17]. We commenced data analysis after seven interviews were completed. The data collection strategy was refined and the process repeated after every few interviews until no new codes were generated in the five subsequent interviews.

Participants were offered a choice between videoconferencing and telephone for the interviews. All opted for videoconferencing and joined in from a nonclinical area. The initial interview schedule (Supporting Information File 2) was developed by NH, JLM and SRB following a literature review and with input from IBD clinician experts and patient representatives. It was piloted with two clinicians, and evolved iteratively throughout the study as new themes were produced. Interviews were audiorecorded and transcribed verbatim. Field notes were made during the interviews to maintain contextual details for data interpretation, for instance lines of questioning that yielded unexpected responses or new themes were noted [13].

### Research team and reflexivity

Interviews were conducted by NH (clinician/PhD student), following her training in the conduct of interviews from JLM and LW (clinicians/researchers with expertise in qualitative interviewing). Participants were informed of the researcher's clinical background and research interest. Researcher reflexivity was acknowledged as an inherent part of the conduct of the study and interpretation of findings. Field notes on potential researcher biases and debrief after each interview for participants to feed back their experience (on the length of the interview, potentially leading questions, amongst others) enabled the researcher to adapt interview techniques prior to subsequent interviews.

### Analysis

Thematic analysis of the transcripts was undertaken using the framework method [18, 19]. A primarily inductive orientation to analysis was adopted, as described by Braun and Clarke [20, 21]. After coding five transcripts, an initial analytical framework was produced, grouping the codes into subthemes and themes, which was applied to subsequent transcripts, using the qualitative data analysis software NVivo (Pro) v.14. This framework evolved and was refined multiple times until no additional codes were generated. Coded data were charted into a matrix allowing comparison of themes within and across cases [18]. Three transcripts were coded independently by two researchers (NH and JLM). Previous studies have shown that co‐coding at least 10% of data improves coding reliability in [22]. Analysed transcripts were not returned to participants for their feedback due to time constraints.

## RESULTS

Twenty‐three HCPs from 13 English and Welsh hospitals were interviewed over a period of 12 months (nine colorectal surgeons, seven gastroenterologists and seven IBD CNSs). The median interview length was 40 (range 17–59) min. All the CNSs had an exclusive IBD practice. Surgeons and gastroenterologists had an interest or expertise in IBD. Table 1 summarizes the participants’ characteristics and duration of interviews.

**TABLE 1:** Participant characteristics and duration of interviews.

Participant number	Role	Level of care provided by participant's unit	Number of years in post as a consultant or an IBD CNS^a^	Duration of interview (min)
HCP01	IBD CNS	Tertiary	6	31
HCP02	Surgeon	Tertiary	1	54
HCP03	Surgeon	Tertiary	20	19
HCP04	Surgeon	Tertiary	2	18
HCP05	Surgeon	Secondary	10	42
HCP06	Surgeon	Secondary	14	17
HCP07	IBD CNS	Tertiary	19	24
HCP08	IBD CNS	Secondary	4	50
HCP09	IBD CNS	Secondary	6	54
HCP10	Gastroenterologist	Tertiary	3	42
HCPINT11	Gastroenterologist	Secondary	25	19
HCPINT12	Gastroenterologist	Secondary	1	27
HCPINT13	Gastroenterologist	Tertiary	5	40
HCPINT14	Gastroenterologist	Tertiary	15	44
HCPINT15	Gastroenterologist	Tertiary	20	59
HCPINT16	Surgeon	Secondary	13	36
HCPINT17	Surgeon	Tertiary	7	39
HCPINT18	Surgeon	Secondary	7	27
HCPINT19	IBD CNS	Tertiary	13	45
HCPINT20	Gastroenterologist	Tertiary	13	32
HCPINT21	IBD CNS	Tertiary	17	48
HCPINT22	Surgeon	Secondary	16	52
HCPINT23	IBD CNS	Secondary	15	54

Abbreviations: CNS, clinical nurse specialist; IBD, inflammatory bowel disease; secondary, secondary care centre for IBD; tertiary, tertiary referral centre for IBD.

^a^ At the time of the interview.

**FIGURE 1:**
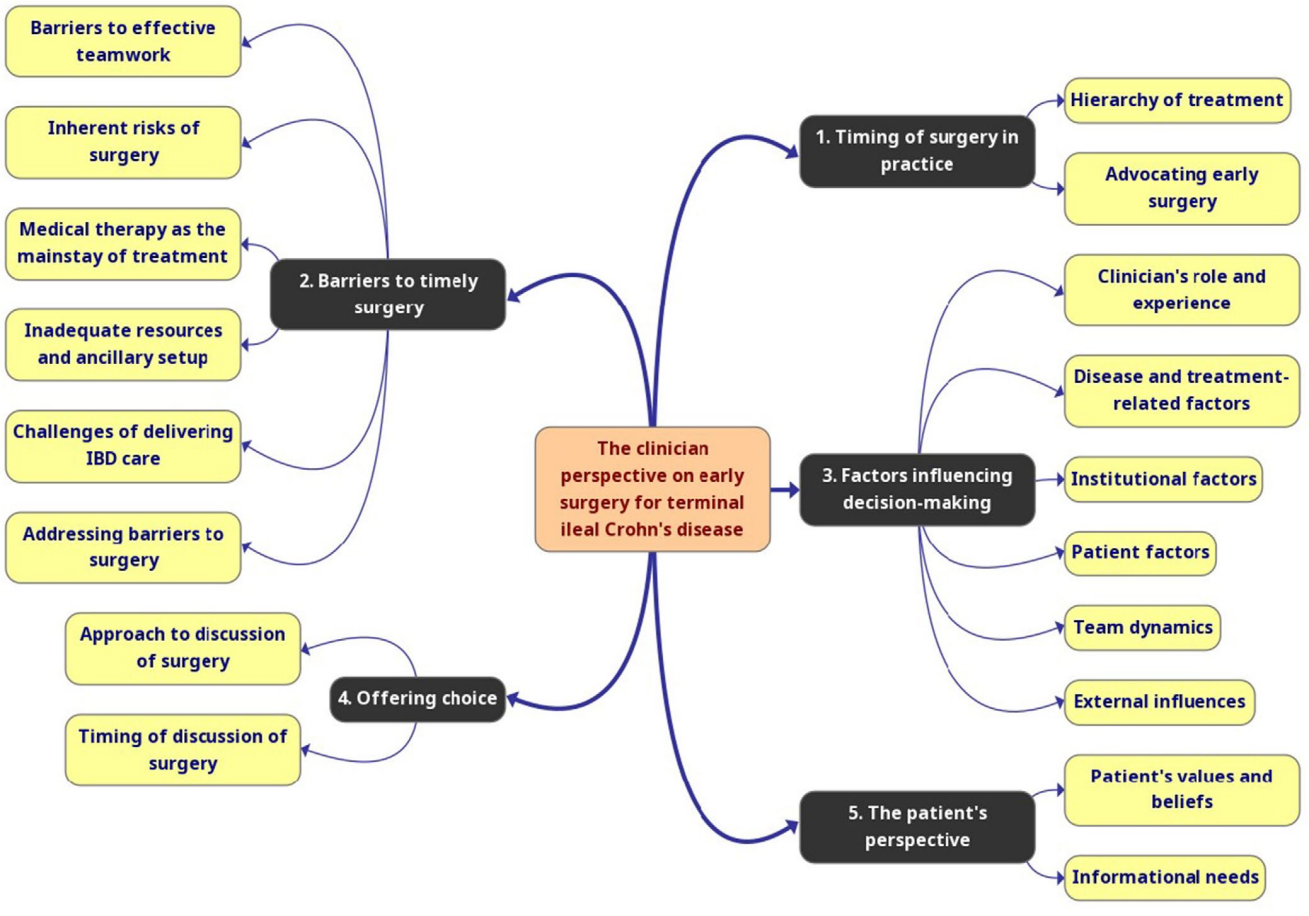
Schematic of themes and corresponding subthemes identified within data. IBD, inflammatory bowel disease.

Data saturation was achieved after the 18th interview and confirmed after the 23rd. Five themes relevant to the research question, with a number of subthemes within each, were identified (Figure 1). These are described below. Tables 2, 3, 4, 5, 6 contain representative quotes for each subtheme.

**TABLE 2:** Theme 1: Timing of surgery in practice.

*Subtheme 1.1: Hierarchy of treatment* ‘We tend to exhaust the medical options before we put them forward for the joint surgical clinic’: HCP01, IBD CBS, tertiary referral centre. ‘Surgery is considered the last resort’: HCP03, surgeon, tertiary referral centre
*Subtheme 1.2: Advocating early surgery* ‘In 50 per cent of patients it [surgery] might cure you, and with possible improvements in surgical intervention, that percentage might get higher. […] If you have medical treatment, you're going to end up with surgery in any case in about 80 per cent of patients.’: HCP03, surgeon, tertiary referral centre ‘[…] then they [patients] finally come to me for an operation, and they're much less well, they've lost a lot of weight, they're malnourished, and you kind of think they'd have been better served with an operation right at the beginning.’: HCP16, surgeon, secondary care ‘The outcomes of surgery have been improved significantly over time.’: HCP17, surgeon, tertiary referral centre

### Theme 1: Timing of surgery in practice (Table 2)

#### Subtheme 1.1: Hierarchy of treatment

Interviewees described a practice of starting medical therapy in patients with newly diagnosed purely inflammatory TI CD. A low threshold for introducing biologicals in a ‘top‐down’ fashion was described. Surgery was the preferred option to manage complications (e.g. fibrostenosis causing prestenotic dilatation) where medical therapy would be futile. In the absence of clear indications for surgery, medical management would continue for as long as possible; a bowel resection was ‘resorted to’ when medical treatment failed.

#### Subtheme 1.2: Advocating early surgery

The benefits of an earlier operation, especially longer‐term remission and the avoidance of long‐term medication, were acknowledged by all interviewees. Surgeons were generally more ardent advocates of early surgery than gastroenterologists and IBD CNSs, highlighting the improvements in surgical techniques and outcomes over time. They discussed the consequences of prolonged medical therapy, including malnourished patients and difficult and riskier operations. Of the 14 participants who discussed the clinical equipoise between medical therapy and surgery for ileocaecal disease, 10 felt that this existed in selected patients with short segment disease and at low risk for recurrence.

### Theme 2: Barriers to timely surgery (Table 3)

**TABLE 3:** Theme 2: Barriers to timely surgery.

*Subtheme 2.1: Barriers to effective teamwork* ‘We don't have a very joined up approach … these cases tend to go to gastroenterology, and they tend to ask for surgical opinion when they feel it's necessary, which is perhaps not the best approach.’: HCP05, surgeon, secondary‐care centre ‘Consultants [gastroenterologists] will make the decision […] and will send a letter to their [surgical] colleague next door. That takes weeks before someone reviews the letters.’: HCP09, IBD CNS, secondary‐care centre
*Subtheme 2.2: Inherent risks of surgery* ‘I suppose I'd have concerns about doing such a big operation and the fact that they may need azathioprine anyway. Should we not just try the azathioprine first, see if that works?’: HCP01, IBD CNS, tertiary referral centre ‘If you're coming to clinic, and you've got somebody like me, who says, look, we can do you an operation, but it's an operation that carries risks. Those risks include a risk to your life, a risk of an anastomotic failure, the risk of a stoma, the risk of a nontrivial complication, and I can't cure you. Or somebody will give you some medicine and we will see if we can settle it down. I think in the first instance, that isn't very balanced, I think most people will go for medicine. I think I'd probably go down the medical route myself.’: HCP16, surgeon, secondary‐care centre ‘[…] they have the operation, and they're home by the end of the week. With medical treatment, regardless of whatever you're [trying], it still takes some time.’: HCP22, surgeon, secondary‐care centre
*Subtheme 2.3: Medical therapy as the mainstay of treatment* ‘I think we feel a lot of these patients would respond to medical treatment. […] because of the experience we've got with biologics and we feel that on the whole they're quite safe and we've got huge experience.’: HCP07, IBD CNS, tertiary referral centre ‘Being creatures of habit, I think that is ingrained in so many physicians, well, I've got five drugs, let me try all of them and see what happens.’: HCP14, gastroenterologist, tertiary referral centre ‘I think sometimes surgery just isn't considered, it's not part of the treatment strategy.’: HCP17, surgeon, tertiary referral centre
*Subtheme 2.4: Inadequate resources and ancillary setup* ‘[…] there's a bit of a wait for two to three months [for a joint clinic] and […] we can start a biologic much quicker than that, within two weeks. […] patients will quickly vote with their feet and say, “I'll go for them [biologics], it's quicker”.’: HCP13, gastroenterologist, tertiary referral centre ‘Waiting lists [for theatre] at the minute are horrendous. […] there are a lot of patients sitting on the waiting list who we're having to start on third‐ or fourth‐line biologics because we know they're not going to get an operation anytime soon.’: HCP19, IBD CNS, tertiary referral centre
*Subtheme 2.5: Challenges of delivering IBD care* ‘There's all the two‐week wait stuff for the cancer work, and if people breach it, we'll jump up and down, but actually with the IBD stuff, there's none of those kinds of benchmarks and targets.’: HCP16, surgeon, secondary‐care centre ‘I think there we have a problem nationally because every hospital doesn't have IBD surgeon.’: HCP14, gastroenterologist, tertiary referral centre ‘It [the IBD MDT] has not been established in a very sustainable way. […] Because we don't have a dedicated pool of surgeons to do IBD, […] the attendance from surgeons is very ad hoc. Unlike, a cancer MDT, where if you didn't have a surgeon or an oncologist […], that would be non‐quorate. […] Whereas you have an IBD MDT, […] you go for several weeks without a surgeon being present […] Some weeks we have an IBD MDT and we don't have a radiologist.’: HCP05, surgeon, secondary‐care centre
*Subtheme 2.6: Addressing barriers to surgery* ‘You're having to attend the cancer MDT and the IBD MDT, you're still dividing your attention. […] the larger hospitals have got dedicated IBD people, I think that will drive care forward for patients.’: HCP05, surgeon, secondary‐care centre ‘Make sure that things like primary outcome of studies is not ‘need for surgery or bowel resection’. […] make sure that studies don't end with surgery as being the failure of the study.’: HCP17, surgeon, tertiary referral centre ‘[…] because our practice has changed that we are getting involved earlier, we're not seeing as much of that late presentation [of Crohn's disease]. […] we moved the MDT a day which means that all four of the IBD interested surgeons could attend […]. That's made a difference.’: HCP18, surgeon, secondary‐care centre

#### Subtheme 2.1: Barriers to effective teamwork

A gastroenterology‐led service was generally described, whereby physicians decide which patients to refer to surgical clinics. Not all centres have joint medical–surgical clinics. There were more reports of inadequate opportunities for the IBD multidisciplinary team (MDT) to work together and a lack of a close working relationship between the medical and surgical IBD teams amongst secondary‐care participants. Generally, tertiary‐care centre surgeons described more active involvement in treatment‐related decision‐making compared with their secondary‐care counterparts.

#### Subtheme 2.2: Inherent risks of surgery

Most HCPs who discussed the risks associated with surgery deem them to significantly outweigh those of medical therapy, making them more cautious about offering surgery when medical options exist. Concerns around postoperative recurrence were voiced more frequently by gastroenterologists and CNSs than by surgeons. Five surgeons viewed the risks of surgery as an acceptable trade‐off when weighed against those of medical therapy.

#### Subtheme 2.3: Medical therapy as the mainstay of treatment

Gastroenterologists and IBD CNSs described their positive experience with biologicals, emphasizing their efficacy, safety, patient‐friendly mode of administration (subcutaneous injections), cost‐effectiveness and the range of options available. These benefits were felt to have led to medical therapy becoming the established norm.

#### Subtheme 2.4: Inadequate resources and ancillary setup

Pressures on resources within the healthcare system impacted the ability of clinicians to facilitate timely surgery. Commonly discussed issues were the long waiting times for elective surgery, the limited availability of joint IBD clinics and time pressures preventing in‐depth discussions about treatment options.

#### Subtheme 2.5: Challenges of delivering IBD care

Many participants mentioned the disparity between cancer care and IBD care in terms of resource allocation and clear benchmarking standards. Cancer surgeries take priority over ‘benign’ resections. A lack of dedicated IBD specialists (especially surgeons) was deemed to compound this problem and have a negative impact on the robustness of surgical decision‐making, especially in smaller centres. The criteria for discussing patients with ileocaecal CD at MDT meetings varied across centres. IBD MDT meetings in some secondary‐care centres were inconsistently attended by surgeons.

#### Subtheme 2.6: Addressing barriers to surgery

**FIGURE 2:**
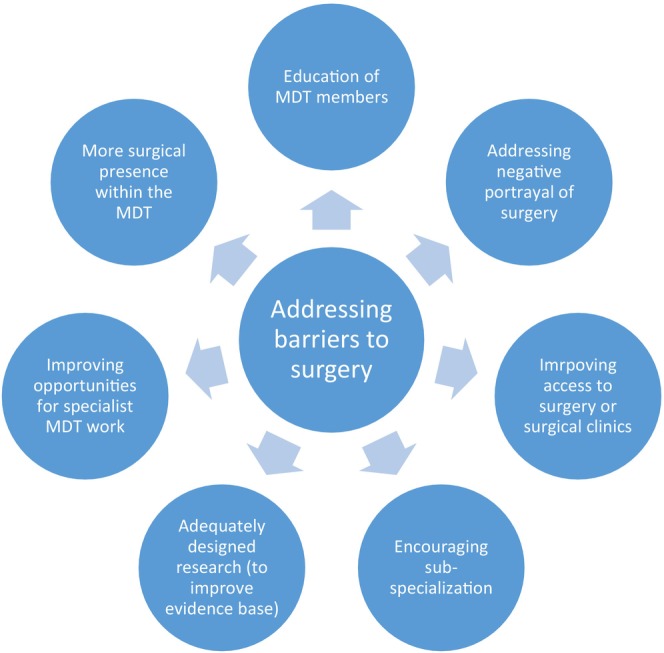
Suggested strategies for addressing barriers to timely surgery. MDT, multidisciplinary team.

Interviewees suggested some strategies to facilitate timely surgery, summarized in Figure 2. These centred on education of the MDT, the creation of opportunities for regular MDT work to enhance the dialogue between the medical and surgical teams, and improved access to surgical or joint clinics. They felt that clinicians should address the negative portrayal of surgery amongst patients through balanced discussions of treatment. Having more IBD subspecialists within the MDT and an increased surgical presence would prompt clinicians to consider surgery sooner. Three HCPs discussed the need for well‐designed studies to improve the quality of the current evidence base comparing early surgery and medical therapy.

### Theme 3: Factors influencing decision‐making (Table 4)

**TABLE 4:** Theme 3: Factors influencing decision‐making.

*Subtheme 3.1: The clinician's role and experience* ‘I don't know enough about the surgery, or I don't feel that I'm best placed to discuss surgery with patients.’: HCP01, IBD CNS, tertiary referral centre ‘I probably perceive the risks of surgery versus outcome very differently to a gastroenterologist.’: HCP04, surgeon, tertiary referral centre ‘There is a substantial proportion of gastroenterologists who would mention surgery as an option, if they mention it at all, just as a sort of passing comment with not much discussion about the risks and the benefits.’: HCP03, surgeon, tertiary referral centre ‘My [general] surgical colleagues will probably dismiss the idea of even considering surgery in someone without an obstruction or an emergency. That is why I will speak to my IBD surgeon.’: HCP14, gastroenterologist, tertiary referral centre
*Subtheme 3.2: Disease and treatment‐related factors* ‘[…] that's probably the group [patients that come in acutely] that gets a bit more of an unbiased opinion from both sides. The patients that come through the elective route, almost exclusively come through to gastroenterology.’: HCP05, surgeon, secondary‐care centre ‘I would only offer early surgery to patients with isolated disease.’: HCP20, gastroenterologist, tertiary referral centre
*Subtheme 3.3: Institutional factors* ‘[My] perception would be that in centres that have a much bigger surgical faculty, probably more people would go for surgery. But we're quite a small DGH, so our surgical focus will often be on the cancers.’: HCP08, IBD CNS, secondary‐care centre ‘The way we objectively assess response, relatively early […] is probably done more robustly […] at a place that is research active than the district general, […] so we probably drive patients through treatment algorithms a bit quicker.’: HCP10, gastroenterologist, tertiary referral centre
*Subtheme 3.4: Patient factors* ‘Even if there is evidence in favour of one or the other, the patient may not want to go down that route.’: HCP03, surgeon, tertiary referral centre
*Subtheme 3.5: Team dynamics* ‘In units where […] there is a disconnect between gastroenterology and surgery […] gastroenterologists see the risk of surgery as higher. […] in units where we work together a lot more closely it's seen as a transition. That you will transition your care between surgery and medicine over the course of your treatment.’: HCP04, surgeon, tertiary referral centre ‘In our trust, the IBD nurses work with the gastroenterologists, and surgeons have their own separate team. Not specifically for IBD, but they have their cancer nurses and we don't have such a close working relationship with the surgeons.’: HCP08, IBD CNS, secondary‐care centre
*Subtheme 3.6: External influences* ‘I think people do take into account evidence but I think people will discount evidence that doesn't fit their paradigm of what treatment entails.’: HCP04, surgeon, tertiary referral centre ‘I think we need more information to be able to take the lead that ileocecal Crohn's disease should be managed top down, i.e. surgery first. I think we're not quite there in terms of our equipoise.’: HCP17, surgeon, tertiary referral centre

#### Subtheme 3.1: The clinician's role and experience

Surgeons generally felt that gastroenterologists had a comparatively more negative view of surgery, which may be reflected in how they discuss it with patients. Gastroenterologists reportedly considered escalation of medical therapy more naturally in their capacity as physicians. Some CNSs felt more comfortable discussing medical options than surgery with patients, limited by their knowledge of the latter. There was consensus that clinicians subspecializing in IBD were likely to be more ‘proactive’ in terms of offering an operation.

#### Subtheme 3.2: Disease‐ and treatment‐related factors

Interviewees discussed the complex interplay of disease‐related factors that shape their management strategy including phenotype, symptoms and endoscopic and biochemical markers. The aim of treatment was reportedly to achieve the biggest improvement in patients' symptoms and quality of life while avoiding treatment complications. Surgeons were more likely to see patients early in the disease course if they presented acutely as an inpatient.

#### Subtheme 3.3: Institutional factors

Participants reported broader influences at an institutional level on their attitude towards the timing of surgery for isolated TI disease. Practice is guided by the department ethos, which in turn is influenced by resource availability, the presence of IBD subspecialists, the size of the IBD surgical team and involvement of the unit in research.

#### Subtheme 3.4: Patient factors

The influence of the patient's age, comorbidities, smoking status and compliance with medical treatment on the decision‐making process were discussed. Patient choice is prioritized over adherence to research evidence or clinician recommendations where equipoise exists.

#### Subtheme 3.5: Team dynamics

A close working relationship between the gastroenterology and surgical team was commonly cited as a key factor in driving decision‐making in favour of earlier surgery.

#### Subtheme 3.6: External influences

Half of the interviewees suggested that their practice was influenced by the evidence base, quoting the LIR!C data; a few discussed the issues with the evidence base that limit its translation into practice. All but two interviewees felt that the cost of treatment did not affect their decision‐making.

### Theme 4: Offering choice (Table 5)

**TABLE 5:** Theme 4: Offering choice.

*Subtheme 4.1: Approach to discussion of surgery* ‘There are definitely a very small proportion of people that might say, I don't want to be on long‐term therapy, I'd probably rather have surgery. I wouldn't like to think that we sway them out of that, but it's possible that we do.’: HCP08, IBD CNS, secondary‐care centre ‘Sometimes, the gastroenterologists have just gone, oh, they'll just nip it out, sort of thing, and actually they're often a bit more surprised when we talk about what actually is involved …. Some of the people are surprised when I tell them it's a general anaesthetic.’: HCP16, surgeon, secondary‐care centre
*Subtheme 4.2: Timing of discussion of surgery* ‘We have a policy in [our unit], which I think is in line with IBD standards, that if there is a need for escalation of medical therapy, […] that surgery should be mentioned to that patient as an option for treatment.’: HCP03, surgeon, tertiary referral centre ‘If you don't bring it [surgery] up early and you bring it up later on down the line, it's a much harder conversation to have because they then are medical therapy obsessed and focused and don't want the surgery.’: HCP19, IBD CNS, tertiary referral centre

#### Subtheme 4.1: Approach to discussion of surgery

Some gastroenterologists and CNSs report being ‘cautious’ bringing up surgery to avoid ‘scaring’ patients early in the course of the disease. Surgeons thought that patients' knowledge of surgery was generally limited prior to their first surgical consultation. HCPs acknowledged their own influence on patients' decision‐making. A team‐based approach was described as the best way of providing a balanced view of medical and surgical options to patients and enabling shared‐decision making.

#### Subtheme 4.2: Timing of discussion of surgery

Most gastroenterologists and CNSs (patients' first points of contact) reportedly mention surgery during the first few consultations but in varying levels of detail. For many, an in‐depth discussion was deemed most appropriate when a switch to a second biological was being considered. Three participants (IBD CNSs) do not routinely mention surgery to newly diagnosed patients. HCPs felt that discussing surgery early on allowed patients to become more accepting of an operation.

### Theme 5: The patient's perspective (Table 6)

**TABLE 6:** Theme 5: The patient's perspective.

*Subtheme 5.1: Patients’ values and beliefs* ‘I would think most people would want medical treatment first, especially if it's really safe medical treatment, for example ustekinumab or vedolizumab.’: HCP10, gastroenterologist, tertiary referral centre ‘Most of the patients would rather opt for medical therapy over surgery. I think that's because of the age of the patients that we see. They're often working, and they want something that they can just carry on with their lives, they don't want to […] have a whole episode where they have to come into hospital and have a recovery period.’: HCP08, IBD CNS, secondary‐care centre ‘That is often their biggest fear […] the thought of waking up with the bag.’: HCP04, surgeon, tertiary referral centre ‘A lot of the people that do grumble along on medical therapy for a long time, and then have surgery, often say, I wish—I should have had that a year earlier, or two years earlier, because it's improved [my] quality of life.’: HCP05, surgeon, secondary‐care centre
*Subtheme 5.2: Informational needs* ‘I feel that it's kind of overwhelming to be told, here are three drugs you've never heard of before, you can't pronounce, and an operation that may involve a stoma.’: HCP10, gastroenterologist, tertiary referral centre ‘It's usually Google that's to blame for patients being hostile to surgery, I think.’ HCP21, IBD CNS, tertiary referral centre

Clinicians shared their perception of patients' preferences, which influence their decision‐making.

#### Subtheme 5.1: Patients’ values and beliefs

There was an overarching feeling that most patients found the risks of medical therapy more acceptable and would prefer to avoid an operation as far as possible. HCPs generally believe that patients find medical therapy easy to administer with minimal disruption to their life. The risks and unknowns of surgery and, especially, of disease recurrence necessitating further operations, were mentioned as deterrents for patients.

The most dreaded risk was felt to be that of a stoma, due to the perceived impact on lifestyle, body image and relationships. HCPs report that patients become more accepting of surgery with disease progression. It was felt that only a minority of patients prefer surgery over medical therapy; these patients are thought to be attracted to the prospect of being drug‐free and having an improved quality of life.

#### Subtheme 5.2: Informational needs

Many nonsurgical participants felt that patients generally preferred to avoid in‐depth discussions about surgery earlier in the course of their disease to avoid feeling overwhelmed. Some HCPs voiced their concerns about the quality of information on online patient forums, which offer a view of surgery skewed by anecdotes of other patients' poor experiences and outcomes.

## DISCUSSION

Clinicians in this study were predominantly in favour of earlier surgery for isolated luminal TI CD, although they report that this does not happen in practice, including their own. These findings are supported by the Crohn's National Confidential Enquiry into Patient Outcome and Death (NCEPOD) audit (2023)—20% of patients with CD experienced a delay in being referred for surgery and 42% waited longer than 18 weeks for surgery [23].

Since the launch of our study, results from two population‐based studies have been published supporting early surgery for CD. They showed that earlier surgery was associated with a reduced need for reoperation and a higher chance of drug‐free remission compared with ongoing medical treatment [24, 25]. Our study is timely, showing that despite the growing body of evidence favouring early surgery there are multiple barriers to its implementation.

There were some variations in opinions and practice amongst clinicians. Participants had different attitudes to the risks of surgery and treatment options seemed to be presented to patients in a way that reflected the clinician's viewpoint. Surgeons felt that surgery was discussed superficially by their medical colleagues, who had a more negative view of its risks and favoured medical therapy due to their positive experience with it. Reports of a disconnect between the medical and surgical teams mostly came from secondary‐care participants, who also more frequently described a shortage of IBD specialist surgeons in their units. This may be because lower‐volume centres are likely to prioritize areas other than IBD care.

Our participants' concerns about the lack of benchmarking standards for the delivery of IBD care are reflected by the findings of the Crohn's NCEPOD audit—only 13% of 138 participating hospitals had local targets for the scheduling of CD surgery [23]. The IBD standards set out recommendations for high‐quality IBD care in the UK, including criteria for referral for MDT discussion and timelines for elective surgery [26]. Our study demonstrates that these standards are not consistently enforced. Some are harder to achieve in the context of wider pressures on NHS resources.

The National Institute for Health and Care Excellence has laid out ‘quality standards’ for IBD care, recommending that surgery is undertaken by a surgeon with experience in IBD surgery [27]. Our participants highlighted the pressure on surgical services to prioritize cancer surgery, thus limiting opportunities for colorectal surgeons to develop a dedicated IBD practice. The need for more IBD specialists—especially IBD surgeons—was a recurring theme in our study, reflecting the national shortage of IBD specialists [28]. Ideally, every centre should have a policy for the care of patients with CD, setting out auditable standards, including targets for surgery. Such changes require leadership from dedicated IBD specialists.

Education of clinicians treating IBD, through regular teamwork, was felt to be key to changing the mindset that surgery represents a failure of medical management and to ensuring that patients receive unbiased information. Teamwork might also help clinicians keep abreast with changes in medical and surgical treatments for localized CD and agree the relative positioning of medical therapy and early surgery locally. Indeed, HCPs' views on their relative positions will now be influenced by the PROFILE study, which reported after the conclusion of our interviews [29]. In this trial, the very early introduction of a ‘top‐down’ approach (infliximab and an immunomodulator) gave substantially better 1‐year outcomes than an accelerated step‐up approach. Conversely, newer techniques, such as the Kono‐S anastomosis, could make an ileocolic resection more appealing if proven to reduce disease recurrence [30].

Limitations of our study include responder bias, primarily attracting participants with an interest in the research topic. The findings are not necessarily generalizable to UK practice or to non‐UK setups. However, participants were from various centres of differing sizes across England and Wales. A range of views are therefore represented. The interviewees were aware of the study team's research interest, which may have somewhat affected their response. Nonetheless this is the first study to provide an in‐depth understanding of the issues underlying the gap between evidence and practice with regards to the management of TI CD in the UK.

Some participants highlighted the problems with the current evidence base that affect its translation into practice. It is unclear when in the disease course surgery should be considered as an alternative to medication. LIR!C only included patients with luminal nonstricturing inflammatory disease, limiting its external validity. More effective and cheaper medical treatments have entered clinical practice since LIR!C concluded [31], and the way in which biological medication is deployed may change in the light of the PROFILE study [29]. This ever‐changing therapeutic landscape makes it difficult to define the equipoise, evidenced by our participants' differing views on the appropriate time to offer surgery. Studies comparing bowel resection and medical therapy at different points during the disease course may be required to address these issues.

For the time being, decisions about the management of ileocaecal CD remain preference sensitive. Therefore, shared decision‐making is essential. Our participants believe that most patients prefer to avoid surgery unless absolutely necessary. It has been shown that patients' preferences for medical or surgical treatment for CD differ from those of their clinicians' [32]. A study exploring the patient perspective on the timing of surgery and acceptability of early surgery for ileocaecal CD is under way (NCT06116604). This will determine whether patients' views align with those of HCPs, and whether their treatment needs are being met.

## CONCLUSION

Surgery is still considered late for the treatment of TI CD. This study provides new insights into the barriers to surgery from a clinician perspective. Further work is required to understand the patient perspective.

## AUTHOR CONTRIBUTIONS

**Nilofer Husnoo:** Conceptualization; investigation; funding acquisition; writing – original draft; methodology; validation; visualization; writing – review and editing; software; formal analysis; project administration; data curation. **Morgan J:** Conceptualization; investigation; methodology; validation; writing – review and editing; software; formal analysis; supervision; project administration. **Lynda Wyld:** Conceptualization; writing – review and editing; methodology; validation; supervision; project administration. **Alan J. Lobo:** Conceptualization; writing – review and editing; supervision; project administration. **Steven R. Brown:** Conceptualization; funding acquisition; validation; formal analysis; supervision; writing – review and editing; project administration.

## FUNDING INFORMATION

This work was supported by a grant from the Sheffield Hospitals Charity (grant reference: 202114) awarded to NH. The funds were used to cover the costs of transcription of the audiorecordings, done by Sterling Transcription Ltd. (https://www.sterlingtranscription.co.uk/).

## CONFLICT OF INTEREST STATEMENT

AJL receives speaker and/or consulting fees for Takeda, BMS, Abbvie, Medtronic, Vifor, Janssen, Sandoz, MSD, Pfizer and Shield Therapeutics.

## ETHICAL APPROVAL

University of Sheffield Research Ethics Committee (reference 044137).

## Supporting information


Supplementary File 1.

Supplementary File 2.

## Data Availability

The interview transcripts cannot be shared publicly for ethical reasons—there is a risk of participants and/or their units being identified. The complete list of coding generated from analysis of the data has been shared (Supporting Information file 1).
