# Supplementary material for: The challenges of implementing earlier surgery for terminal ileal Crohn's disease—A qualitative study of the clinician's perspective
Source: Colorectal Dis. 2025 Feb 11;27(2):e70027. doi: 10.1111/codi.70027 (PMC11814344; doi:10.1111/codi.70027)
Supplement: Supplementary file 1 [file CODI-27-0-s001.docx]

Interviews with healthcare professionals – coding tree

| **Themes** | **Subthemes** | **Codes (I)** | **Codes (II)** |
| --- | --- | --- | --- |
| 1. Timing of surgery in practice | Advocating early surgery | Equipoise |  |
|  |  | Evolving practice |  |
|  |  | Rationale for advocating early surgery | Alternative to escalation of medical therapy |
|  |  |  | Benefits of early surgery  - Avoiding long term medication or immunosuppression  - Beneficial in selected patients  - Better quality of life  -Forward planning  -Improvement in surgical care  -Instant improvement or cure in limited disease  -Longer term remission  -Minimally invasive and low morbidity  -Technically easier to perform |
|  |  | Consequences of delayed surgery |  |
|  |  | Drawbacks of medical therapy | Delaying surgery - more complicated, higher recurrence rate |
|  |  |  | Impact on QoL |
|  |  |  | Incomplete remission (prolonged symptoms) |
|  |  |  | Lack of response or poor tolerance |
|  |  |  | Long term effects unknown |
|  |  |  | Long-term immunosuppression or need for medication |
|  |  |  | Mode of administration and its impact on patient's QoL |
|  |  |  | Regular monitoring |
|  |  |  | Side effects |
|  |  |  | Slower onset of effect, steroids as a bridge |
|  |  |  | Treatment escalation |
|  |  |  |  |
|  | Hierarchy of treatment | Medical treatment as the norm | Role of medical therapy  - accepted and established practice  - an option alongside surgery  - Initial therapy of choice  - Therapy of choice in mild or purely inflammatory disease |
|  |  | The appeal of medical therapy | Avoiding surgery |
|  |  |  | Cheap |
|  |  |  | Effectiveness |
|  |  |  | Good experience and easy to initiate |
|  |  |  | Less invasive |
|  |  |  | Mode of administration becoming more patient-friendly |
|  |  |  | Multiple options, expanding armamentarium |
|  |  | Risks of surgery | Diarrhoea |
|  |  |  | Failure to improve symptoms or disappointing outcomes |
|  |  |  | Medical therapy may have worked |
|  |  |  | Non curative |
|  |  |  | Reasons to delay or avoid surgery |
|  |  |  | Side effects and complications |
|  |  | Role of surgery | Complications of disease |
|  |  |  | Considered after trial of at least some medical therapy |
|  |  |  | Exhaustion of medical options or last resort |
|  |  |  | Medical therapy risky, futile or not tolerated |
|  |  |  | Obstructive symptoms or abdominal pain |
|  |  |  | Patient request |
|  |  |  | Primary therapy for short segment or severe disease |
|  |  | Surgery considered late |  |
|  |  |  |  |
| 2. Barriers to timely surgery |  |  |  |
|  | Addressing barriers to surgery | Addressing negative portrayal of or stigma around surgery |  |
|  |  | Adequately designed research |  |
|  |  | Education of MDT members |  |
|  |  | Encouraging sub-specialisation |  |
|  |  | Improving access to surgery or surgical clinics, earlier referral |  |
|  |  | Improving opportunities for specialist MDT work |  |
|  |  | Improving peri and post-operative care |  |
|  |  | Improving timely and accurate diagnosis |  |
|  |  | More surgical presence within the IBD MDT |  |
|  |  |  |  |
|  | Barriers to effective teamwork | Disconnect between medical and surgical team |  |
|  |  | Lack of opportunities for MDT work |  |
|  | Challenges of delivering IBD care | Benign surgery not prioritised |  |
|  |  | Clinician's role, experience and expertise |  |
|  |  | Lack of dedicated IBD surgeons or specialists |  |
|  |  | Variations in standard of IBD care | Benchmarking standards not well-defined |
|  |  |  | Delays in referring to specialist services |
|  |  |  | Education and expertise |
|  |  |  | Good standards of care |
|  |  |  | IBD vs cancer care |
|  |  |  | Reasons for suboptimal care |
|  |  |  | Specialist vs generalist approach |
|  | Inadequate resources and ancillary setup | Pressure on resources and delays in accessing services |  |
|  |  | Suboptimal peri and post-operative management |  |
|  | Inherent risks of surgery | Historical association with poor outcomes |  |
|  |  | Non-curative nature, not seen as definitive measure |  |
|  |  | Perception that risks of surgery outweigh those of medical therapy |  |
|  | Medical therapy as the mainstay of treatment | Clinicians not used to considering surgery |  |
|  |  | Good experience with medical therapy |  |
|  |  | Perception of surgery being a failure of medical therapy |  |
|  |  | Prolonged medical therapy - unoptimised patient |  |
| 3. Factors influencing decision-making | Clinician's role and experience | Attitude towards risk |  |
|  |  | Clinician's role and ethos |  |
|  |  | Specialist (more proactive) vs generalist |  |
|  | Disease and treatment related factors | Disease-related factors | Disease genetics |
|  |  |  | Disease phenotype |
|  |  |  | Duration of disease or symptoms |
|  |  |  | Involvement of other sites |
|  |  |  | Length of segment |
|  |  |  | Likelihood of needing surgery |
|  |  |  | Likelihood of recurrence or needing post-op treatment |
|  |  |  | Likelihood of responding to medical therapy |
|  |  |  | Mode of presentation (elective vs acutely unwell) |
|  |  |  | Objective measures (endoscopy, imaging) |
|  |  |  | Presence of complications |
|  |  |  | Previous treatment and response to treatment |
|  |  |  | Severe or aggressive vs mild disease |
|  |  |  | Symptoms and impact of disease on patients |
|  | Treatment-related factors | Efficacy of treatment and mechanism of action |  |
|  |  | Mode of administration |  |
|  |  | Recognising the limitations of treatment |  |
|  |  | Risks, complications or morbidity |  |
|  |  | Consideration of treatment optimisation strategies | Optimisation of follow up and post-operative care |
|  |  |  | Optimisation of medical treatment |
|  |  |  | Patient engagement and good rapport |
|  |  |  | Patient optimisation for surgery |
|  | External influences | Costs | Costs of treatment |
|  |  |  | Costs to patient |
|  |  | Evidence-base and guidelines or policy | Problems with evidence base |
|  | Institutional factors | Research active unit |  |
|  |  | Resources |  |
|  |  | Variation in approach between institutions |  |
|  | Patient factors | Age |  |
|  |  | Attitude to risk and ability to cope with treatment |  |
|  |  | BMI |  |
|  |  | Co-morbidities or fitness for surgery |  |
|  |  | Compliance with treatment |  |
|  |  | Life events |  |
|  |  | Nutritional and physiological status |  |
|  |  | Patient choice |  |
|  |  | Smoking |  |
|  |  | Surgical history |  |
|  | Team dynamics | Collaboration or disconnect amongst MDT members |  |
|  |  | Department ethos |  |
|  |  | MDT setup and involvement | Convergent or divergent values between gastroenterologists and surgeons |
|  |  |  | Criteria for and timing of involvement |
|  |  |  | IBD patients only managed by IBD specialists |
|  |  |  | Opportunities for team-working  - Barriers to team work  - Consistent or inconsistent involvement of surgeons  - Examples of clinical activities enabling teamwork |
|  |  |  | Role of the MDT  - Achieving consensus  - Addressing uncertainties  - Education of colleagues within MDT  - Instilling confidence into patients |
|  |  |  | Similarities or variations within team |
|  |  |  | Specific roles of individual team members  - Active or leading role vs passive  - Gastroenterology-led service  - Role of the IBD CNS |
|  |  |  | Working relationship between team members |
|  |  | Medical colleagues' approach or proactivity |  |
| 4. Offering choice | Approach to discussion of surgery | Approach reflecting preference for initial medical therapy |  |
|  |  | Challenges involved in discussing surgery |  |
|  |  | Consistency of approach within team |  |
|  |  | Agreement amongst colleagues |  |
|  |  | Variation according to role and expertise |  |
|  |  | Decision-making style | Influence of patient’s age |
|  |  |  | Patient's ability to understand information |
|  |  |  | Preference for shared decision-making |
|  |  | Depth of discussion around surgery |  |
|  |  | Influence of clinician's approach on patient |  |
|  |  | Vision for ideal approach | Discussion by appropriate clinician |
|  |  |  | Team-based approach |
|  | Timing of discussion of surgery | Early discussion |  |
|  |  | Influence of timing on patient's decision-making |  |
|  |  | Later discussion |  |
|  |  | Shared view on appropriate timing |  |
|  |  | Tailored to individual needs |  |
|  |  |  |  |
| 5. The patient's perspective | Informational needs | Existing knowledge of treatment options |  |
|  |  | Preferences for volume of information |  |
|  |  | Sources of information | Charity organisation |
|  |  |  | Clinician |
|  |  |  | Lack of resources on surgery (including early surgery) for patients |
|  |  |  | Medical literature |
|  |  |  | Other patients and relatives |
|  |  |  | Patient support groups or peer support |
|  |  |  | Problems with patients' sources of information |
|  |  |  | Social media or online forums |
|  | Patient's values and beliefs | Attitude to risks |  |
|  |  | General fear of an operation | Experience of surgical pathway or inpatient stay |
|  |  |  | Risk of a stoma  -Influence of age on views  -Associated stigma  -Body image  - Clinician's approach to discussion  - Cultural background  - Fear of unknown and unpredictability  -Feels unnatural  -Gender  -Impact on relationships and sex life  -Others' experience  -perceived impact on lifestyle  -Sexual function and fertility  -Social media  - Factors mitigating negative views: Body positivity movement, Clinician's communication style, debunking misconceptions, - Fashion brands catering for people with a stoma, - In-depth and considered discussion, rapport with clinician, - Online information about surgery improving, - Low risk of a permanent stoma, -Stoma nurses' input, Peers’ experiences, -Presence of IBD nurse specialist  -Public figures or role models |
|  |  |  | Risks and unknowns  -Body image  -Denial – time required to accept  -Impact on QoL, family and work  -Lack of control and uncertainty  -Mortality  -Non-curative or risk of recurrence  -Ongoing treatment and-surveillance-post-operatively  -Perceived as last resort measure  -Poor surgical outcome  -Recovery  -Risks of surgery |
|  |  |  | The appeal of surgery  -Being off medication  -‘Cure’ in isolated disease, complete removal  -Hindsight bias  -Instant improvement in symptoms |
|  |  | General preference for medical therapy | Risks more 'benign' |
|  |  |  | Trade-off preferences |
|  |  |  | Examples where medical therapy less acceptable |
|  |  | Influence of lived experience of disease | Impact of disease or treatment on life |
|  |  |  | Side effects of treatment and mode of administration |
|  |  |  | Symptoms consistent with objective findings |
|  |  |  |  |
| **Additional topics explored to identify attributes to design discrete choice experiment** | | | |
| *Outcomes and treatment goals* | *Convergent or divergent patient and clinician values* | *Factors considered when choosing treatment* |  |
|  |  | *Outcomes* |  |
|  |  | *Treatment choice* |  |
|  |  | *Views on surgery* |  |
|  | *Outcomes important to clinicians* | *Avoiding complications or side effects* |  |
|  |  | *Cost* |  |
|  |  | *Lack of symptoms or symptom control* |  |
|  |  | *Being drug-free* |  |
|  |  | *Objective, measurable outcomes* |  |
|  |  | *Quality of life and function* |  |
|  |  | *Remission or lack of recurrence* |  |
|  | *Outcomes important to patients from a clinician perspective* | *Avoiding risks or complications of treatment* |  |
|  |  | *Avoiding a stoma* |  |
|  |  | *Avoiding surgery* |  |
|  |  | *Disease remission* |  |
|  |  | *Minimally invasive surgery vs open* |  |
|  |  | *Not taking medication* |  |
|  |  | *Quality of life and regaining control* |  |
|  |  | *Symptom resolution* |  |
