# Supplementary material for: The challenges of implementing earlier surgery for terminal ileal Crohn's disease—A qualitative study of the clinician's perspective
Source: Colorectal Dis. 2025 Feb 11;27(2):e70027. doi: 10.1111/codi.70027 (PMC11814344; doi:10.1111/codi.70027)
Supplement: Supplementary file 2 [file CODI-27-0-s002.docx]

## Healthcare Professionals Interview schedule

**The questions relate to patients with isolated terminal ileal or ileocaecal Crohn’s disease (L1 phenotype) without prior surgery. This excludes patients with very clear indications for surgery, for example symptomatic fibrotic stricture, or with very clear indications for medical therapy, for example multiple diseased segments involving a significant length of bowel.**

1. **What is your general approach to managing a patient with a new diagnosis who does not respond fully to steroids or relapses quickly off steroids?**

Prompts: What factors would make you consider immune modulators?

What factors would make you consider biological therapy?

What would make you consider offering an ileocaecal resection?

1. **You have mentioned some of the factors that guide your management. What are the other factors that influence your choice of therapy in this scenario?**

Prompts: *Patient factors*

Age of patient

Co-morbidities

Smoking status

Patient choice

*Disease factors*

Duration of disease

Duration of previous treatments

Number of courses of steroids

High risk disease features (e.g. perianal disease, fistulating disease)

Risk of relapse or disease recurrence

Development of complications/disease behaviour

Extra-intestinal manifestations

*Organisational and other factors*

Guidelines, including local guidelines

Costs of treatment

IBD MDT meeting discussions

Availability of joint clinics

Research evidence

Own experience

For gastroenterologists and surgeons: Nurse specialist’s input

For gastroenterologists & IBD CNS: Ease of involving surgical colleagues and relationship with them

For surgeons: Gastroenterology colleagues’ threshold for involving surgeons in the management

1. **In your view, what are the risks and benefits of continuing medical therapy and those of performing a bowel resection in this patient at this stage in their treatment?**

Prompts: Side effects & safety profile of medical therapy

Quality of life

Duration of benefit

Why would you not offer surgery at this stage? (Complications of surgery, recurrence, acceptability to patient)

Is the likelihood of medically treated patients needing surgery in next 12 -24 months a consideration?

Is the likelihood of surgically treated patients needing antiTNF in next 12 -24 months a consideration?

1. **When should a bowel resection be offered to a patient in this cohort? Where does it fit in in the management algorithm?**

Prompts: Complications of disease

Frequent relapses, hospitalisations

Steroid dependent/ steroid refractory disease

Once immunosuppressants have been trialled for a given period of time

Failure of medical therapy

Unacceptable side effects

Patient’s choice

1. **When would you first discuss surgery with this patient, and how would you approach this discussion?**

Prompts: In what circumstances would an in-depth discussion of surgery be had and formally offered as a treatment option?

What criteria would lead you to refer a patient to the IBD joint clinic for consideration of surgery?

1. **In scenarios where you feel that both ongoing medical therapy and an ileocaecal resection are valid options, what role does the patient play in decision-making?**

Prompts How is that input obtained?

1. **How do you think patients feel about an ileocaecal resection for limited TI disease?**
2. **Any additional comments**
